# Screening for domestic abuse and its relationship with demographic variables among elderly individuals referred to primary health care centers of Shiraz in 2018

**DOI:** 10.1186/s12877-020-01667-9

**Published:** 2020-08-17

**Authors:** Maryam Hazrati, Maryam Mashayekh, Nasrin Sharifi, Seyedeh Ameneh Motalebi

**Affiliations:** 1 Deputy Ministry of Nursing Affairs, Ministry of Health and Medical Education, Tehran, Iran; 2 Community Based Psychiatric Care, Research Center, , Shiraz University of Medical Sciences (SUMS), Shiraz, Iran; 3 Fatemeh Zahra School of Nursing and Midwifery, Shiraz University of Medical Sciences, Shiraz, Iran; 4 Epidemiology, School of Nursing and Midwifery, Shiraz University of Medical Sciences, Shiraz, Iran; 5 Social Determinants of Health Research Center, Research Institute for Prevention of Non-Communicable Diseases, Qazvin University of Medical Sciences, Qazvin, Iran

**Keywords:** Elderly, Misbehavior, Screening

## Abstract

**Background:**

Elder abuse is an important public health problem. The present study was aimed to determine the rate of domestic abuse and its relationship with demographic characteristics among elderly people referred to Primary Health Care (PHC) centers in Shiraz, Iran, 2018.

**Methods:**

This descriptive and cross-sectional study was conducted on 400 older people aged 60 years old and above who referred to 22 PHC centers of Shiraz. The data were collected using demographic characteristics questionnaire, Katz index, the domestic elder abuse questionnaire, and elder neglect checklist through face-to-face interview and observation methods. Backward linear regression model was used for analyzing the data.

**Results:**

The results indicated that 52.5% of the participants were female and 51.8% aged 60–69 years old. A total of 159 cases (39.8%) reflected at least one form of elder abuse or neglect. The results indicated that 21% of the participants (*n* = 84) were abused by their own children. Care neglect was the most reported form (42.8%), followed by psychological abuse (41.3%), emotional neglect (38.8%), and financial abuse (34.3%). The most common types of neglect were motion limitations (25%) followed by the dental problems (23.8%). The results also showed a significant relationship between domestic elder abuse and level of income (*p* = 0.017), having a house (*p* = 0.028), type of perpetrator (< 0.001), and insurance status (*p* = 0.027).

**Conclusions:**

The results revealed a considerable rate of domestic abuse against elderly people, causing a serious risk for their health and security.

## Background

Population aging is a global phenomenon [1, 2] caused by improvement of healthcare, reduction of birth rate, and increase in life expectancy [3]. To date, there is 650 million people aged 60 years or over across the world, which is expected to reach 2 billion by 2050 [4]. Older population is growing in Iran, as well [5]. According to the United Nations in 2002, based on the assumption of a moderate growth rate, people over 60 would comprise nearly 25% of Iran’s population by 2040–2050 [6]. One of the consequences of population aging is elder abuse and neglect. In fact, technological advances, financial welfare, and changing family structures have caused to hurt the relationships between older people and their family members [7]. Elder abuse is a significant global phenomenon experienced by many individuals across the world [4]. To date, no specific definition exists for elder abuse. Based on the World Health Organization (WHO), elder abuse has been defined as “a single or repeated act or lack of appropriate action occurring within any relationship where there is an expectation of trust which causes harm, distress to an older person” [8, 9]. Such behaviors may include psychological, emotional, physical, financial, or sexual abuses, authority deprivation, ostracism, neglect, and refraining from providing a healthy environment [10–13].

### Each type of abuse has a specific definition as follows

**Psychological or emotional abuse is an** inappropriate reaction to individuals’ emotions and feelings such as verbal threat, and insult.

**Physical abuse** is any form of physical force or punishment causing bodily injury, physical impairment or a behavior pattern that assaults all physical and sexual aspects of the elderly causing pain and injury. Physical abuse includes hitting, beating, pushing, burning, and kicking.

**Financial abuse** is illegal or improper use of an elder’s funds, assets or property and it includes forging the elder’s signature and the unauthorized use of their checks.

**Sexual abuse** is any kind of sexual contact without a person’s consent. Such contact may involve unwanted touching and various forms of sexual harassment and rape.

**Authority deprivation** is another form of elder abuse that occurs when the elders are deprived of the right to choose, decision-making, and comment.

**Ostracism** is the act of expelling an older individual from his/her home and rejection by the family members.

Neglect is the most common type of elder abuse [9]. It occurs when caregivers (family members, social security staff, and private caregivers) fail to fulfill their duties towards a dependent elderly person [4] or when a nurse or caregiver are not able to meet the needs of an elderly person properly. Neglect can take different forms including physical, financial, psychological, and self-neglect. Physical neglect means failing to attend to an elderly person’s nutrition, medical, and hygienic needs. Financial neglect means neglecting to manage an elderly person’s assets. Abandoning affectional needs of an elderly person or prevention their involvement in social activities and communicating is referred to psychological neglect. Self-neglect involves elderly people with disabilities who fail to meet their own essential physical, psychological or social needs [11].

The rates of elder abuse are likely to be underestimates as many cases of them are not reported. The results of a previous research showed that 3–4% of the elderly suffer from different types of abuse [14]. Yon et al. (2019) estimated the global rate of elder abuse to be 15.7%. Accordingly, one out of six elderly people was involved in elder abuse [15].

Domestic abuse is one of the most common types of domestic violence, which is the manifestation of deprivation from human rights [7]. Most cases of elder abuse are perpetrated by the spouse (25%) and the older son of the family (50%) [16–19]. The risk factors of elder abuse include age, sex (female), physical, mental, and social problems, familial problems, loneliness, suffering from dementia, social isolation, drug abuse by elderly individuals or nurses, and family history of violence [3].

In Iran, a limited number of studies have been conducted on elder abuse ( [7, 8, 20, 21], and). Khanlary (2004) reported that retired elderly residing in Karaj, Iran experienced at least one type of abuse and 9.7% of them introduced their children as perpetrators [20]. Heravi Karimoie (2002) also reported that the rate of elder abuse was 9.5% among retired elderly people in Tehran, Iran [7]. Overall, the necessity of further investigation in this area is warranted. The American Medical Association has also emphasized for elder abuse screening in order to diagnose and prevent different types of this health issue [22].

In most studies conducted on domestic abuse in Iran, the data were collected by questionnaires ([7, 8, 20, 21, 23], and). However, considering the nature of domestic elder abuse, it seems that the quantitative method could not present a precise picture of the elderly population’s status. Hence, screening technique may yield more accurate information about this social issue. Therefore, the present study was aimed to determine the rate of elder abuse in elderly people referred to the primary healthcare centers of Shiraz, Iran.

## Methods

This descriptive and cross-sectional study was conducted in 2018 for screening the elder abuse. The convenient sampling method was used to include 400 eligible older people referring to the PHC centers in Shiraz, Southern Iran, for receiving primary health care services. Sampling was done from 22 comprehensive health centers covered by Shohaday-e-Enghelab and Shohaday-e-Valfajr health centers. Based on a similar study performed by Morovati Sharifabaid et al. [23] in Yazd in 2016, considering *p* = 0.63 (the rate of abuse), α = 0.05, d = 0.05, and non-response rate = 0.10, and using the following formula, sample size was finalized at 400 subjects.

$$ N=\frac{{Z_{1-\alpha /2}}^2\times p\left(1-p\right)}{d^2} $$

Participants were required to be 60 years or older and able to communicate verbally to be included in the study. Participants who reported having psychiatric problems (e.g., Alzheimer’s disease) or were unwillingness to take part in the study were excluded from the study.

Data were collected from 21st April to September 2018 by a nurse researcher who was trained for conducting interviews and completing the questionnaires. In order to encourage the participants to cooperate, their blood pressure was measured for free. Screening of domestic abuse was done through an interview and observation.

### Interview

The data were collected by four questionnaires consisted of demographic characteristics (gender, age, marital status, education level, etc.), Mini-mental state examination to identify elderly people with cognitive impairment who are prone to be abused. Katz index of independence for determining the degree of dependence to perform the activities of daily living [24], and the domestic elder abuse questionnaire that was designed and validated by Heravi in 2009. It consists of 49 items with eight domains including care neglect (11 items), psychological abuse (8 items), physical abuse (4 items), financial abuse (6 items) authority deprivation (10 items), rejection (4 items), financial neglect (4 items), and emotional neglect (2 items) [21].

**Mini-mental state examination** is a commonly used set of questions for screening cognitive function and finding individuals suffering from dementia. A score of 7 to 10 suggests normal cognitive status, 4–6 indicates moderate cognitive impairment, and 1–3 suggests severe cognitive impairment [25].

### Observation

After the interviews, all participants were assessed regarding the signs of neglect and abuse using the domestic elder abuse checklist. This checklist was designed by Hervai-Karimooi et al. (2013) with 11 items appears to be a promising tool for detecting neglect among elders in different settings by health care providers or researchers [26]. In this study, because of ethical issues and impossibility of home visit, four items that were related to the neglect in providing healthy environment were not assessed. The researcher completed this checklist through observational technique. The participants who had the signs of physical injury (bruise on the face, ecchymosis on the body, wounds, and scratches) with no logical reasons were referred to the healthcare centers for the further assessments.

#### Statistical analysis

The data were analyzed by Statistical Package for Social Sciences, version 22.0 (SPSS Inc., Chicago, Illinois). Demographic variables were described using frequencies and percentages for categorical variables and mean and standard deviations for continuous variables. Backward linear regression model was used to determine the predictors of domestic elder abuse. Significance level was set at *p* ≤ 0.05.

#### Ethical consideration

The study was approved by the Ethics Committee of Shiraz University of Medical Sciences, Shiraz, Iran (IR.SUM.REC.1396.S1045). All participants signed written informed consent after taking some information about the objectives, procedures, potential benefits, and drawbacks of the study. Furthermore, they were instructed that their participation is voluntary and their information will be kept confidentially.

## Results

This study was conducted on 400 older adults including 210 females (52.5%) and 189 males (47.3%). The highest frequency was related to the 60–69 age group (51.8%). Most of the participants were married (*n* = 296, 74%), illiterate (*n* = 213, 53.3%), had 4–7 children (*n* = 197, 49.3%) and pensions (*n* = 211, 52.8%). Additionally, 351 participants (87.8%) lived in their own houses and 268 ones (67.0%) lived with their spouses. Besides, 221 participants (55.3%) suffered from non-communicable chronic diseases and 382 ones (95.5%) were covered by insurance.

The results indicated that 21% of the participants (*n* = 84) were abused by their own children. Moreover, 46.3% of the older participants (*n* = 185) obtained 7–10 scores in the mini-mental state examination indicating a good cognitive status, 37% (*n* = 148) gained 4–6 scores representing a moderate cognitive status, and 16.8% (*n* = 67) obtained 1–3 scores indicating a bad cognitive status. Furthermore, the majority of the participants were independent in doing their daily activities.

We found that the most and least domestic abuse were care neglect (42.8%) and ostracism (4.3%), respectively. The frequency and percentage of the domestic abuse subscales were presented in Table 1.

**Table 1:** The frequency of domestic elder abuse subscales

Elder abuse subscales	Yes Frequency (%)	No Frequency (%)
Emotional neglect	155 (38.8)	245 (61.2)
Care neglect	171 (42.8)	229 (57.2)
Financial neglect	115 (28.8)	285 (71.3)
Authority deprival	118 (29.5)	282 (70.5)
Psychological abuse	165 (41.3)	235 (58.8)
Ostracism	17 (4.3)	383 (95.8)
Physical abuse	21 (5.3)	379 (94.8)
Financial abuse	137 (34.3)	263 (65.8)

According to the results of the current study, at least one type of neglect was observed in 159 of 400 (39.8%) assessed cases. The most common types of neglect were motion limitations (25%) followed by the dental problems (23.8%) (Table 2).

**Table 2:** The frequency of neglect among the participants based on the observational elder neglect checklist

Observational neglect	Yes Frequency (%)	No Frequency (%)
Inappropriate clothes	27 (6.8)	373 (93.3)
Smelly body and clothes	15 (3.8)	385 (96.3)
Dirty and untidy hair	12 (3)	388 (97)
Long and dirty nails	12 (3)	388 (97)
Dental problems	95 (23.8)	305 (76.3)
Bedsore	6 (1.5)	394 (98.5)
Motion limitation	100 (25)	300 (75)

The relationship between demographic characteristics and various types of domestic abuse was explored in this study. The results revealed significant relationships between different types of domestic abuse and all demographic features, except for gender, age, marital status, number of children, and occupation. The relationships between demographic characteristics and various types of domestic abuse have been depicted in Table 3.

**Table 3:** The relationship between the demographic characteristics of the elderly respondents and the types of domestic elder abuse

Subscales	Demographic characteristics	Standardize Coefficients	t	*p*-value
Emotional neglect	(Constant)	_	6.350	< 0.001
	Income	0.105	2.362	0.19
	Living arrangement	− 0.105	−2.447	0.015
	Perpetrator	−0.476	− 10.708	< 0.001
	Insurance	− 0.088	−2.061	0.040
	Cognitive status	0.110	2.547	0.015
Care neglect	(Constant)	_	5.580	0.001
	Income	0.110	2.461	0.014
	House ownership	0.102	2.238	0.026
	Living arrangement	−0.117	−2.659	< 0.001
	Perpetrator	− 0.478	−11.226	< 0.001
	Cognitive status	0.122	2.796	0.005
Financial neglect	(Constant)	_	5.040	< 0.001
	Education level	−0.139	−3.020	0.003
	Income	0.192	4.135	< 0.001
	Perpetrator	−0.347	−7.652	< 0.001
Authority deprival	(Constant)	_	4.704	< 0.001
	House ownership	0.141	2.937	0.004
	Health status	−0.112	−2.331	0.020
	Perpetrator	−0.238	−4.932	< 0.001
Psychological abuse	(Constant)	_	4.887	< 0.001
	Employment	0.085	1.816	0.070
	Perpetrator	−0.389	−8.264	< 0.001
	Insurance	−0.088	−1.863	0.063
Physical abuse	(Constant)	_	0.877	0.381
	Living arrangement	0.144	2.954	0.003
	Perpetrator	−0.221	−4.535	< 0.001
Financial abuse	(Constant)	_	5.040	< 0.001
	Living arrangement	0.100	5.040	0.034
	Health status	0.097	2.060	0.040
	Perpetrator	−0.379	−7.952	< 0.001
	Insurance	−0.099	−2.074	0.039
Ostracism	(Constant)	_	0.912	0.057
	House ownership	0.111	2.245	0.025
	Perpetrator	−0.183	−3.706	< 0.001
Total Scale	(Constant)	_	7.580	< 0.001
	Education level	−0.072	−1.759	0.079
	Income	0.103	2.391	0.017
	House ownership	0.092	2.210	0.028
	Perpetrator	−0.574	−14.066	< 0.001
	Insurance	−0.089	−2.214	0.027

## Discussion

In this study, the most common misbehaviors were care neglect (42.8%), psychological abuse (41.3%), emotional neglect (38.8%), and financial abuse (34.3%). These findings are consistent with other studies in Iranian populations [27–30]. For instance, Mohebbi et al. (2013) [29] and Nasiri et al. (2014) estimated the rate of psychological abuse and care neglect to be 53.3 and 59.8%, respectively [8]. Furthermore, Heravi Karimooi (2013) found that the most prevalent types of elder abuse were emotional neglect (74.45%) and psychological abuse (62.22%) [28]. However, Kissal in Turkey (2011) reported the rates of psychological abuse, neglect, and financial abuse were 9.4, 8.2, and 2.1%, respectively [9]. These inconsistencies in the results from different studies usually have been arisen from the trend of industrialization, economic challenges, and increased inflation rate in recent years, which have declined children’s supportive capabilities. Furthermore, the incidence of psychological abuse is under the pressures created by economic hardship [30].

In this study, care neglect was the most prevalent type of neglect. Similarly, Keyghobadi et al. (2014) reported that the highest frequency was related to emotional neglect (69%) and care neglect (52%) among older adult residing in Sabzevar, Iran [30]. Chalise and Basnet (2017) also stated that the highest frequency was related to neglect (35.4%) in Nepal [31]. Considering these results, it can be concluded that neglect is the most common type of misbehavior in most countries regardless of their cultures and traditions. Neglect can be manifested through intentional or unintentional refusal or failure in providing physical or psychological care or refrain from giving them food, water, or medications [8]. In the current study, domestic neglect consisted of inattention, lack of personal or telephone contacts, refrain from supplying the needs and cleaning them, and bank’s affairs.

Psychological abuse are often secretly performed kinds of abuse [32]. In the present study, the highest frequency was related to psychological abuse after care neglect. In the same line, Atri et al. (2013) reported that psychological abuse (91%) was the highest frequency abuse among elderly people living in Tabriz, Iran [33]. Psychological abuse also showed the highest frequency (86.10%) in the study by Khanlary et al. (2014) in Karaj [20]. However, Lacher et al. (2016) showed that the rate of psychological abuse was 47% in Sweden [34]. This inconsistencies might be due to the differences between western and eastern culture. For example, in traditional eastern societies, particularly Iran, parents being involved in making major decisions for their children such as a selection of spouse or living place and children respect to the parental decisions. Therefore, even a slight disagreement with older people’s opinion is considered to be disrespect and emotional misbehavior. However, such disagreements are not considered disrespectful in the Western cultures [35].

In this study, authority deprivation (29.5%, *n* = 118) was the fourth most prevalent elder abuse. Even though, the majority of the study participants lived in their own houses (87.8%) however, 29.5% of them experienced authority deprivation due to high dependence rate related to physical and mental illnesses, lack of sufficient income, and relatives’ expectations to share their properties. However, Piri et al. (2018) found that authority deprivation (68.5%) was the first most prevalent domestic elder abuse among elderly women residing in Tehran, Iran [36]. In the current study, authority deprivation were consisted of making elderly people aware of the important news, depriving them of using their properties, and depriving them of having contact with their families and friends.

In this study, the lowest frequency was related to ostracism (4.3%), which might be attributed to the fact that 87.8% of the participants lived in their own houses and they do not allow their children and relatives to interfere in their assets. This finding is supported by the results of Heravi Karimoei et al. (2012) and Piri et al. (2018) who reported the rate of ostracism 11.2 and 3.7% among Iranian older adults, respectively [7, 36].

The findings of the present study revealed a significant relationship between all types of domestic abuse and the perpetrators. The perpetrators were mainly the participants’ children (21%). This result might be the fact that those dealing with the loss of a spouse live with their children and they are highly dependent due to physical and mental disabilities. However, children do not recognize their conditions and abuse them either intentionally or unintentionally. Chalise and Basnet (2017) also conducted a study in Nepal and reported a higher rate of abuse by their family members among elderly individuals with health problems [31].

The results of the current study showed a significant association between lower income and being abused. The living income of the majority of the participants of this study was pension that was not sufficient and some of them even bore their children’s expenditures. Likewise, the results of a previous study showed a significant relationship between older individuals’ physical and economic dependence and being abused, especially when caretakers have to bear the older peoples’ expenses [37]. Furthermore, most participants of this study were female and housewife. Elderly women are more financially, mentally, and socially dependent on their acquaintances. Agha Noori et al. (2012) also stated that in the most of developing countries, including Iran, elderly women have a lower economic and social status than elderly men. Thus, they are more likely to be abused and neglected. These results have been demonstrated in the numerous studies [38–43].

One of the most common risk factors significantly associated with victimization was cognitive impairment in the previous studies [44, 45]. Fang (2018) disclosed that elderly people with dementia were more prone to be abused compared to their counterparts [46]. Furthermore, the risk of death due to abuse and neglect was found to be higher among elderly people with high levels of cognitive disorders [47]. In the current study, most of the participants were in the 60–69 age group and they obtained acceptable cognitive scores. However, 16.8% of them got lower than normal scores, which might be attributed to illiteracy, lack of awareness about the date and some life events, and some degrees of amnesia exposed faced them to some misbehaviors such as emotional and care neglect.

Although 95.5% of the participants of this study was under health insurance coverage, but their insurance and health status were significantly associated with the domestic abuse. This might be the fact that, in Iran, health insurance does not cover all of the costs associated with having an illness. So, the older people with a poor economic status and under health insurance more likely not to benefit from private healthcare services. This might have negative impacts on the participants’ health status and so, it is considered as a risk factor for abuse among older people who most of them suffer from non-communicable diseases.

### Strength and limitations

As a strength, in the present study, domestic abuse screening was conducted using both interview and observation methods.

While this study offers preliminary insights into the rate of domestic elder abuse and its related factors, it is not without limitations. One limitation of the present study was the adoption of a convenience sampling procedure, which may limit the generalizability of the research findings. The sample of this study was community-dwelling elderly adults, and so, it may be difficult to generalize the results to the institutionalized elderly people. Furthermore, the cross-sectional design of the study may restrict the ability to detect any causal relationships among the variables. Moreover, some elderly adults were unwilling to report abuse due to feelings of shame, however after making the trust, they talked about this issue. The final limitation of this study was the impossibility of visiting the living environment of older adults however, it is suggested for the future studies to observe older people in their living environment for better evaluation of the neglect and elder abuse.

## Conclusions

Findings suggest that the rate of elder abuse among Iranian older adults are in high level. The results also showed that the care and emotional neglect and psychological abuse were the most frequent elder abuse in Iran. In addition, education, income, housing, living arrangement, health status, insurance status, perpetrator, and cognitive status were associated factors with various types of domestic elder abuse.

### Implications

Given the fast increasing Iranian older adults, preventive interventions should be considered for managing the elder abuse. On a practical level, the role of nurses’ and physicians’ in prevention and diagnosis of the elder abuse should not be ignored. Community health nurses play an important role in helping the older adults and their family by necessary preventive interventions to reduce elder abuse. To this end, future studies are needed to use both quantitative and qualitative methods to explore the real situation of elderly abuse and associated factors.

## Supplementary information

**Additional file 1.**

## Data Availability

All data generated or analysed during this study are included in this published article [supplementary file: SPSS file].

## Abbreviations

PHC: Primary Health Care
WHO: World Health Organization
